# Supplementary material for: Matched asymptotic solution for crease nucleation in soft solids
Source: Nat Commun. 2018 Feb 5;9:496. doi: 10.1038/s41467-018-02979-6 (PMC5799209; doi:10.1038/s41467-018-02979-6)
Supplement: Supplementary file 1 — Supplementary Information [file 41467_2018_2979_MOESM1_ESM.pdf]

SUPPLEMENTARY FIGURE 1

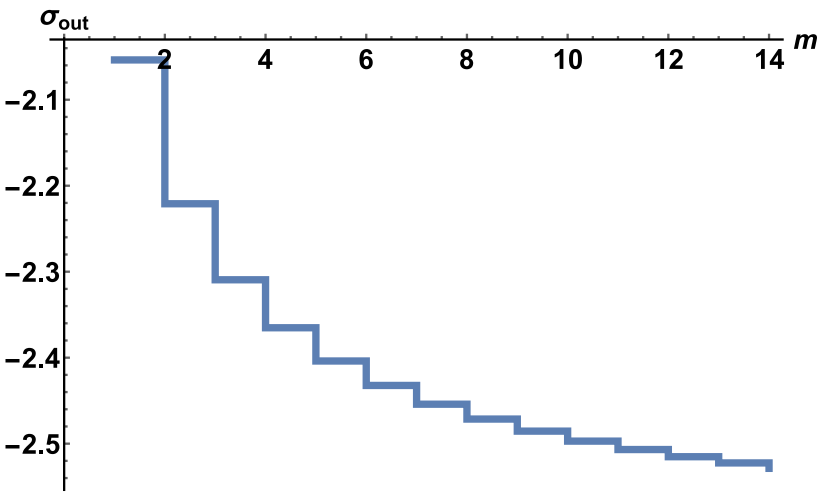

Supplementary Figure 1: Dispersion curve. Marginal stability curve from Eq. (12) showing the critical compressive stress σ_{out} versus the circumferential mode m.

SUPPLEMENTARY FIGURE 2

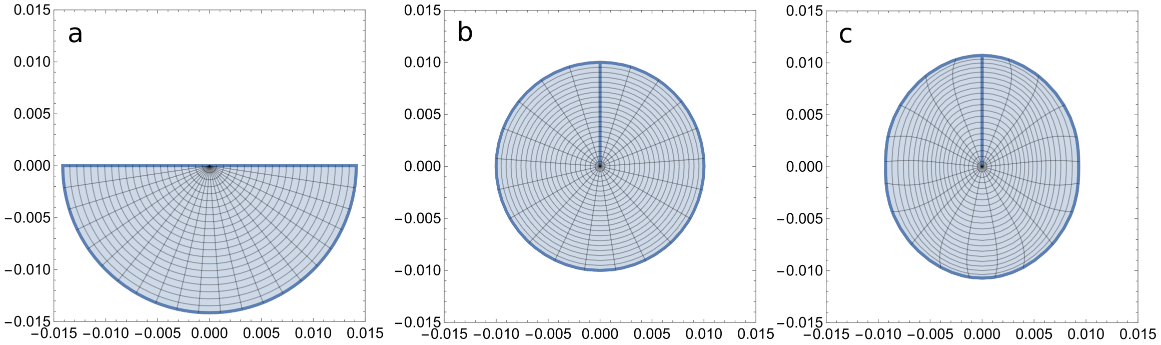

Supplementary Figure 2: Perturbative inner solution. Morphological transitions described by the asymptotic expansion of the inner solution, from an undeformed half circle of radius R_c around the nucleation point (A), to a full circle in the homogeneously deformed state (B), and to the elliptical shape beyond the creasing threshold (C). The parameters are $R_c = 0.014$ and $\epsilon = 0.0015$.

SUPPLEMENTARY FIGURE 3

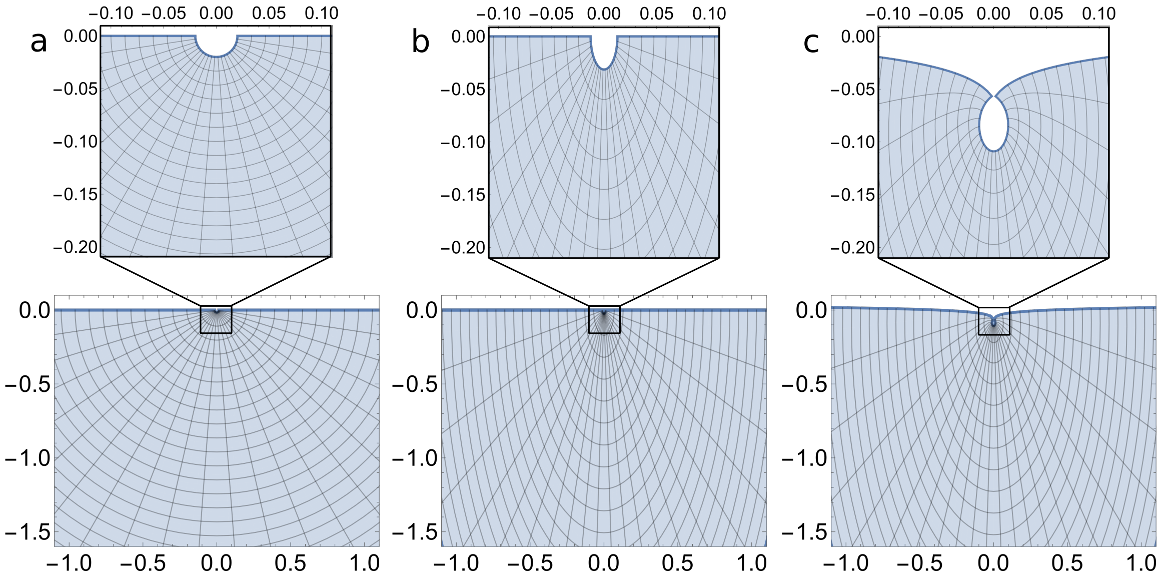

Supplementary Figure 3: Perturbative outer solution. Morphological transitions described by the asymptotic expansion of the outer solution, from an undeformed half-space where a circle of radius 0.02 is removed around the nucleation point (A), to a homogeneously compressed state (B), and to the formation of an elliptical cavity through incipient self-contact beyond the creasing threshold (C).

SUPPLEMENTARY FIGURE 4

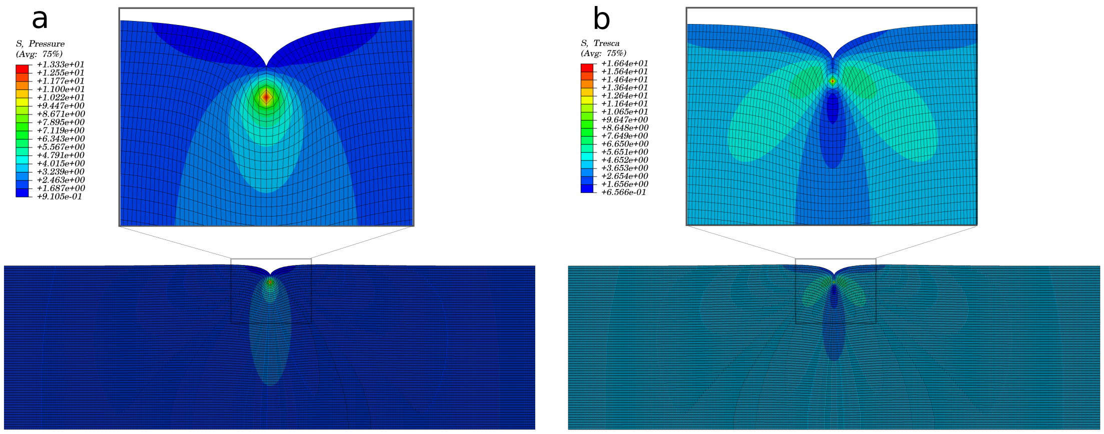

Supplementary Figure 4: Numerical results on stress distribution. Contour plots of the pressure field (left) and the Tresca equivalent stress (right) resulting from the mixed finite element simulation in Figure 3 of the main article. The insets show the numerical solutions in proximity of the self-contacting domain.

SUPPLEMENTARY NOTE 1

Mathematical solution of the incremental problem. Let the inner solution be confined in the spatial domain given by $0 \leq r \leq r_c$, in which r_c is small compared to any characteristic length in a finite size problem. The Neumann boundary condition $\sigma_{rr}(r_c) = \sigma^{out} = \mu\left(\lambda_x^2 - \lambda_x^{-2}\right)$ applies so that the horizontal force balances the uniform horizontal traction exerted by the side walls. Since the self-contact is a unilateral constraint only permitting a compressive hoop stress, it must be assumed in the following that $\sigma^{out} \leq 0$. It is important to highlight that this boundary condition corresponds to the application of a dead load, since this applied traction only exists in the spatial configuration, not having any material counterpart. In order to investigate the local stability of the homogeneous inner solution, the theory of incremental deformation superposed over finite strains is then applied.

Let $\delta\mathbf{x} = [u(r,\theta), v(r,\theta)]^T$ be an incremental deformation vector with respect to the inner creased solution, i.e $|\delta\mathbf{x}| << r_c$, with radial and tangential components u and v, respectively. In particular, writing its spatial gradient $\mathbf{\Gamma}$ as:

$$\mathbf{\Gamma} = \text{grad}(\delta\mathbf{x}) = \begin{bmatrix} u_{,r} & \frac{u_{,\theta}-v}{r} \\ v_{,r} & \frac{v_{,\theta}+u}{r} \end{bmatrix} \tag{1}$$

the incompressibility condition becomes:

$$\text{tr}\mathbf{\Gamma} = u_{,r} + \frac{v_{,\theta}+u}{r} = 0 \tag{2}$$

By standard Taylor expansions around the inner homogeneous solution, the components of the push-forward $\delta\mathbf{S}$ of the incremental Piola-Kirchoff stress tensor read:

$$\delta S_{ji} = A_{jikl}\Gamma_{lk} + p_{in}\Gamma_{ji} - \delta p_{in}\delta_{ji}; \quad \text{with} \quad (\mathrm{i,j,k,l}) = (\mathrm{r},\theta); \tag{3}$$

where δp_{in} is the increment of the hydrostatic pressure, δ_{ji} is the Kronecker delta and A_{jikl} are the elastic instantaneous moduli. For a neo-Hookean material, it can be easily checked that $A_{jikl} = \mu\lambda_j^2\delta_{jk}\delta_{il}$ where $\lambda_r = 1/\sqrt{2}$ and $\lambda_\theta = \sqrt{2}$ are the radial and hoop stretches of the creased solution, respectively.

This incremental boundary value problem is complemented by the equilibrium equation:

$$\text{div}\delta\mathbf{S} = \mathbf{0} \tag{4}$$

with boundary conditions at the free surface given by:

$$\delta S_{rr} = \delta\sigma_{rr} = \frac{d\sigma_{rr}}{dr}u = \mu\frac{3u}{2r_c}; \qquad \delta S_{r\theta} = 0 \quad \text{at } \mathrm{r} = \mathrm{r_c} \tag{5}$$

where $\delta\sigma_{rr}$ is the increment of the external traction caused by the perturbation of the interface. The boundedness of the incremental displacement and stress fields at the crease origin $r = 0$ must finally be enforced for the sake of physical compatibility.

Imposing a sinusoidal perturbation in the hoop direction respecting the expected inversion symmetry over the x axis, a solution is sough by separating the variables as $u(r,\theta) = U(r)\cos(m\theta)$, $v(r,\theta) = V(r)\sin(m\theta)$, where m is the integer circumferential wavenumber. After some standard manipulations of the incremental equations, the boundary value problem is rewritten as a fourth-order ordinary differential equation on $U = U(r)$ as:

$$\begin{aligned} r\left(r\left(r\left(rU'''' + 6U'''\right) - 5\left(m^2-1\right)U''\right)\right. \\ \left. -\left(5m^2+1\right)U'\right) + \left(4m^4 - 5m^2 + 1\right)U = 0 \end{aligned} \tag{6}$$

By imposing physical compatibility at the origin, the solution of (6) is given by:

$$U = \epsilon\left(\frac{r}{r_c}\right)^\alpha + \epsilon c_1\left(\frac{r}{r_c}\right)^\beta; \tag{7}$$

with ϵ being the small amplitude of the incremental perturbation, i.e. $|\epsilon/r_c| << 1$, and

$$\begin{aligned} \alpha &= \sqrt{\tfrac{2+5m^2+m\sqrt{9m^2+40}}{2}}; \\ \beta &= \sqrt{\tfrac{2+5m^2-m\sqrt{9m^2+40}}{2}}; \end{aligned} \tag{8}$$

expressing the two bounded eigensolutions of the incremental problem. The two boundary conditions at $r = r_c$ in (5) read:

$$r\left((\mu + 2\sigma^{out})\mathrm{U}^{'} + \mathrm{r}(\mu)\mathrm{U}^{''}\right) - \left(m^2 - 1\right)(2\sigma^{out} - \mu)\mathrm{U} = 0; \tag{9}$$

$$\begin{aligned}&\mathrm{r}\left(\left(\mu + 2m^2(\sigma^{out} - 3\mu)\right)\mathrm{U}^{'} + \mu\mathrm{r}\left(\mathrm{r}\mathrm{U}^{'''} + 4\mathrm{U}^{''}\right)\right) + \\ &\mu\left(4m^2 - 1\right)\mathrm{U} = 0.\end{aligned} \tag{10}$$

Substituting (7) into (9), if $m > 1$ the constant c_1 is found equal to:

$$c_1 = -\frac{\mu\left(m^2 - 1 + \alpha^2\right) + 2\left(1 - m^2 + \alpha\right)\sigma^{out}}{\mu\left(m^2 - 1 + \beta^2\right) + 2\left(1 - m^2 + \beta\right)\sigma^{out}}. \tag{11}$$

whilst $c_1 = 0$ for $m = 1$.
Using Eqs. (7, 11) into Eq. (10) finally gives the dispersion relation expressing the curves of marginal stability of the homogeneous inner solution for $m > 1$, as follows:

$$\begin{aligned}&m^2\left(\mu^2\left(\alpha^2 + \alpha(7\beta - 3) + \beta^2 - 3\beta + 5\right)\right. \\ &-2\mu\sigma^{out}\left((\alpha + \beta)^2 + \alpha + \beta + 10\right) + 4(\sigma^{out})^2\Big) \\ &+(\alpha + 1)(\beta + 1)\mu(\alpha((\beta - 1)\mu + 2\sigma^{out}) \\ &-\beta\mu + 2\beta\sigma^{out} + \mu) \\ &-2m^4\left(3\mu^2 + 2(\sigma^{out})^2 - 7\mu\sigma^{out}\right) = 0\end{aligned} \tag{12}$$

For $m = 1$, the incremental problem is found to be always stable. Conversely, Eq.(12) admits one real negative root for σ^{out}/μ at varying m (the positive must be discarded since it is not allowed by the self-contact constraint). In particular, increasing circumferential mode m the magnitude of the corresponding critical compressive stress increases, as shown in Supplementary Figure 1. Accordingly, it is found that the creased solution loses its incremental stability for the following value of critical compressive stress at the critical mode $m = 2$:

$$\frac{\sigma_{xx}^{out}}{\mu} = \lambda_x^2 - \lambda_x^{-2} = \frac{\left(1 - 3\sqrt{5} + \sqrt{18 - 2\sqrt{5}} - 2\sqrt{26\sqrt{5} - \sqrt{62\sqrt{5} + 1038} + 28}\right)}{8} \simeq -2.053696; \tag{13}$$

corresponding to the *critical stretch* λ_x^{cr} *for crease nucleation* given by:

$$\begin{aligned}\lambda_x^{cr} = \tfrac{1}{4}\Bigg(&1 - 3\sqrt{5} + 2\sqrt{\tfrac{1}{2}\left(9 - \sqrt{5}\right)} - 2\sqrt{26\sqrt{5} - \sqrt{62\sqrt{5} + 1038} + 28} + \\ &\sqrt{\left(-3\sqrt{5} + \sqrt{18 - 2\sqrt{5}} - 2\sqrt{26\sqrt{5} - \sqrt{62\sqrt{5} + 1038} + 28} + 1\right)^2 + 256}\Bigg)^{1/2} \simeq 0.637554.\end{aligned} \tag{14}$$

Eq. (14) is the first analytic insight of the critical stretch for crease nucleation, being in excellent agreement with previous numerical investigations, reporting a value in the range $0.63 - 0.643$. Deriving an analytic prediction of the creasing threshold allows to identify the physical mechanism triggering nucleation, i.e. the loss of uniqueness of the scale-free inner solution. Moreover, the inner deformation is a universal solution in finite elasticity, meaning that it can be supported in equilibrium for every isotropic material by suitable surface tractions alone. Thus, the creasing threshold specific to a different constitutive equation can be calculated by solving the corresponding inner incremental problem. This work somehow confirms the validity the previously obtained numerical results. Indeed, it is important to highlight that creasing follows a subcritical transition, that is characterized by a high sensitivity to imperfections. Moreover, this transition lacks an energy barrier, so that an infinite degeneracy of creased states is available beyond the critical threshold. In order to capture this discontinuity of the solution beyond the threshold, the mentioned numerical methods employ artificial regularisations, such as a damped pseudo-dynamics and/or the introduction of an energy term at the free surface. Even if such regularising effects are taken as small as allowed for leading the simulations to a successful end, they somehow guide the numerical search in a very particular subspace of solutions. Moreover, the subcritical nature of the bifurcation makes it difficult to perform a rigorous convergence analysis of the regularised numerical problem to the original one, whilst decreasing the intensity of such stabilizing effects.

SUPPLEMENTARY NOTE 2

Matching between the inner and the outer solutions. The perturbed displacement fields of the inner and outer solutions are found to match in proximity of the self-contacting inner domain. This can be checked analytically by transforming the corresponding expressions in polar coordinates, as illustrated in Supplementary Figures 2, 3.
The inner solution is depicted in Supplementary Figure 2, showing that an undeformed half circle around the nucleation point becomes a creased domain whose boundary transforms to an ellipse. This transition towards an ellipse-shaped domain allows matching with the perturbed outer solution, as depicted in Supplementary Figure 3. Indeed, the corresponding near-field displacement fields create an elliptic cavity after removing an undeformed half circle out of the reference configuration. Moreover, the vertical displacement u_y becomes much bigger than the horizontal one u_x (see Eqs. 17 and 18 in the article), showing the incipient formation of a cusped profile at the free surface.
The matching is similarly proved for the pressure fields. In particular, the incremental pressure field $\bar{p}$ for the singularly perturbed outer solution is given by:

$$\bar{p} = -\frac{2a_0\mu\left(\lambda_x^8 - 1\right)y}{\lambda_x^2\left(x^2 + y^2\right)} \tag{15}$$

In polar coordinates, the previous expression indicates that $\bar{p}$ goes like $\cos\theta\ r_c/r$, proving the required finite correction in proximity of the inner solution, i.e. for $r \sim O(r_c)$. However, in such a range the incremental fields become of the same order of the zero-th order terms. Thus, the corresponding perturbative approximation locally becomes ill-posed and a direct matching cannot be provided.

SUPPLEMENTARY NOTE 3

Mixed finite element simulations. A block with unit thickness L and width 8L has been discretized using a structured mesh made of 17600 hybrid, four-node bilinear elements $CPE4H$, setting a large displacement formulation. Self-contact at the free surface is implemented allowing a tangential frictionless contact based on a finite sliding algorithm not allowing overdisclosure, dealing with a self-contact length much smaller than the block size.
Previous numerical works investigated crease nucleation either inserting a small crease-like defect on the reference mesh or applying an axial compression to the material beyond the Biot threshold for forming a single crease, and then gradually removing the strain up to the critical value where the crease disappears. Even if they report similar stretch thresholds for the crease nucleation, both methods have important drawbacks for the nonlinear elastic solution: the former introduces an artificial material singularity, thus never displays the homogeneous outer solution before creasing in the whole domain; whilst the latter cannot ensure that the creased branch reached jumping from the Biot threshold is effectively the same of the initial bifurcation branch at the nucleation threshold. In order to overcome such important limitations, crease nucleation is studied here by applying both a little down-ward nodal force F in the middle of the free surface, setting an intensity $0.0025\mu\ L^2$, where μ is bulk modulus, and an axial compressive stretch up on the block sides, up to a weakly nonlinear creasing regime at $\lambda_x = 0.62$. The nodal force is then completely removed whilst keeping the stretch as constant.
Creasing nucleation and morphology are finally studied by gradually and completely removing the axial compression whilst keeping a zero nodal force. An adaptive, iterative increment algorithm is set for both nodal force and applied stretch, starting with a maximum step of 0.01 and a minimum of 10^{-12}. Since creasing occurs after a subcritical bifurcation, an automatic stabilization scheme with a constant damping factor γ is implemented in the direct solver using a Full Newton technique.

Supplementary Figure 4 further depicts the contour plots of the hydrostatic pressure field p (a) and the Tresca equivalent stress (b) in the creased block. In particular the resulting pressure field in proximity of the creased domain has the same spatial distribution predicted by Eq.(15).
